# Supplementary material for: Complex‐centric proteome profiling by SEC‐SWATH‐MS
Source: Mol Syst Biol. 2019 Jan 14;15(1):e8438. doi: 10.15252/msb.20188438 (PMC6346213; doi:10.15252/msb.20188438)
Supplement: Supplementary file 8 — Dataset EV7 [file MSB-15-e8438-s008.zip › feature_plots_string/O95721.pdf]

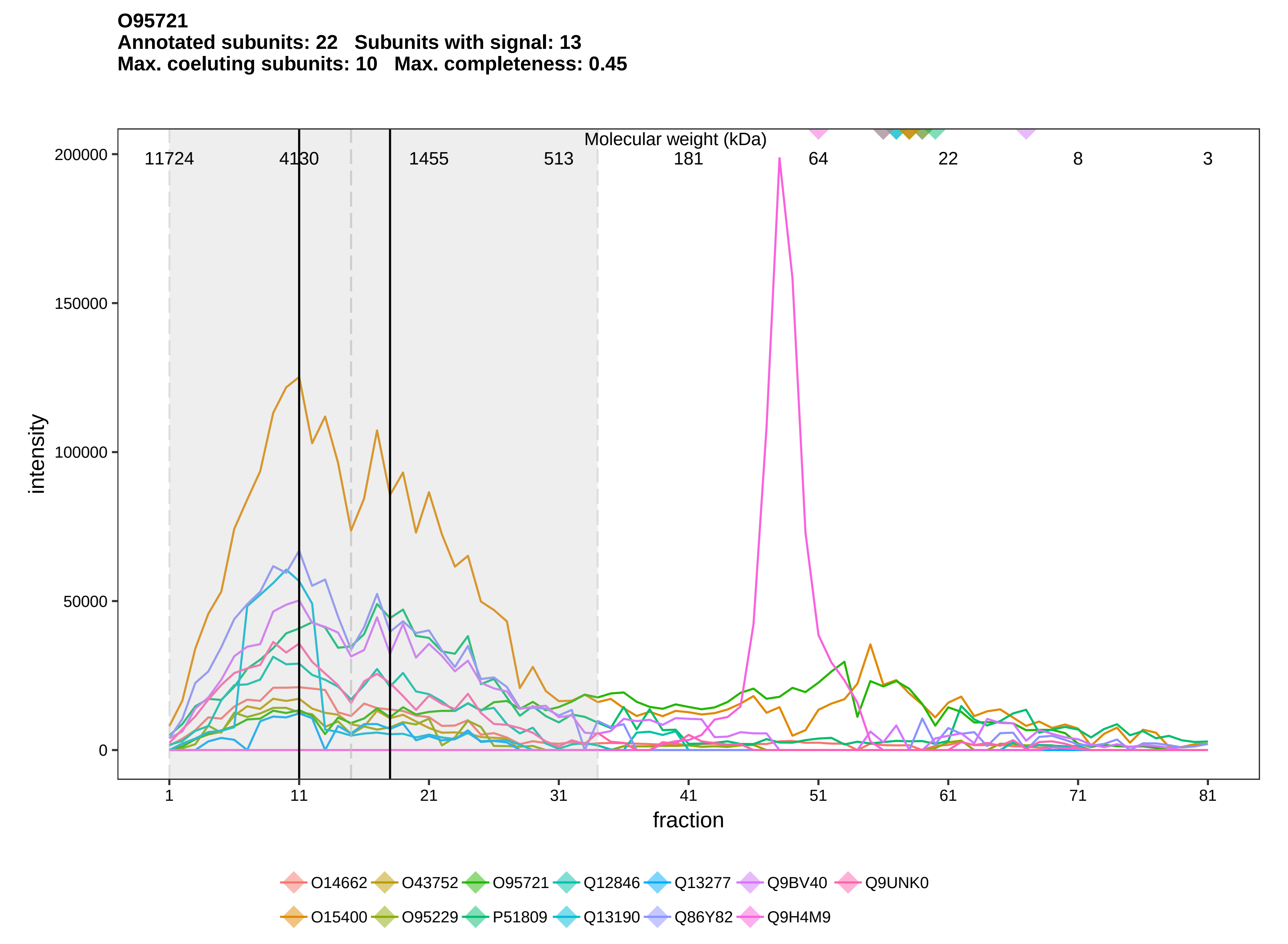
O95721
Annotated subunits: 22   Subunits with signal: 13
Max. coeluting subunits: 10   Max. completeness: 0.45
Molecular weight (kDa)
11724
4130
1455
513
181
64
22
8
3
200000
150000
100000
50000
0
intensity
1
11
21
31
41
51
61
71
81
fraction
O14662
O43752
O95721
Q12846
Q13277
Q9BV40
Q9UNK0
O15400
O95229
P51809
Q13190
Q86Y82
Q9H4M9
